# In Vitro Anticancer Potential of Eugenol on Oral Cancer Cell Lines: A Systematic Review

**DOI:** 10.21315/mjms2024.31.5.2

**Published:** 2024-10-08

**Authors:** Shafa Nafisa Wulan, Jamas Ari Anggraini, Wahyu Hidayat

**Affiliations:** 1 Undergraduate Dentistry Study Program, Faculty of Dentistry, Universitas Padjadjaran, Indonesia; 2 Department of Oral Biology, Faculty of Dentistry, Universitas Padjadjaran, Indonesia; 3 Department of Oral Medicine, Faculty of Dentistry, Universitas Padjadjaran, Indonesia

**Keywords:** eugenol, oral cancer, oral squamous cell carcinoma, anticancer, in vitro, chemopreventive

## Abstract

Oral cancer is one of the most common types of cancer and has become a global health concern. Oral squamous cell carcinoma (OSCC) is the most prevalent form of oral cancer worldwide. Eugenol, an aromatic phenolic compound, exhibits various pharmacological activities, including anticancer effects. Several studies have reported the anticancer activity of eugenol against OSCC via different pathways. However, no current review has discussed the extent of eugenol anticancer research on oral cancer cell lines using in vitro studies. This systematic review aimed to discuss the anticancer potential of eugenol against oral cancer cell lines in vitro. Articles were selected from PubMed, ScienceDirect, SpringerLink and EBSCOhost databases based on specified inclusion and exclusion criteria. Additional articles were identified through manual hand searching. The selection process followed PRISMA guidelines. A risk-of-bias assessment was performed to evaluate the reliability and relevance of the in vitro studies. Thirteen articles with high-quality results were assessed and analysed for further investigation. These studies investigated the ability of eugenol to induce cell death through apoptotic and non-apoptotic pathways, inhibit cell proliferation and affect oxidative stress, contributing to cell death in several oral cancer cell lines. Therefore, eugenol is a potential anticancer agent for OSCC, as it exhibited anticancer activity in oral cancer cell lines in vitro studies.

## Introduction

Oral cancer is one of the most common types of cancer and has become a global health concern. According to the Global Cancer Observatory in 2020, the incidence of lip and oral cancers exceeded 370,000 cases (1, 2). Asia has the highest global incidence of lip and oral cancers, accounting for 65.8% of cases with a mortality rate of 74% (1). Data on national oral cancer cases in Indonesia, reported by Cheong et al. (3), indicates that Indonesia has the highest incidence of oral cancer among Southeast Asian countries, with 5,329 of 18,071 cases.

Over 90% of oral cancers are oral squamous cell carcinomas (OSCCs) (4–8). OSCC is a malignant tumour originating from the stratified squamous epithelium of the oral cavity (9). Currently, OSCC has a high mortality rate of approximately 50% (6, 10–13), and the 5-year survival rate is predicted to be less than 50% (12–14). Previous reports indicate that OSCC accounts for 2% of all cancers in women and 3% in men (11), with the incidence in males being approximately twice that in females (11). OSCC may develop on the lower lip, floor of the mouth, ventral or lateral regions of the tongue, gingiva, buccal mucosa, retromolar region, tonsils or lateral soft palate (4, 7, 15, 16).

Tobacco and alcohol are significant causes of OSCC owing to their high risk of contributing to oral cancer (6, 11, 17). More than 90% of OSCC cases are associated with tobacco consumption (17). Alcohol consumption also contributes to the development of oral cancer (11, 17, 18). Other factors contributing to OSCC include HPV, betel quid chewing and poor nutritional intake (11, 17, 19). Oral cancer progresses due to the loss of cell cycle regulation, leading to increased proliferation and decreased apoptosis, followed by enhanced tumour cell activity towards invasion and metastasis (11). Furthermore, the development of OSCC is correlated with oncogene activation and the inactivation of tumour suppressor genes (11, 15).

Eugenol is an aromatic compound of the phenolic group with an oily consistency, transparent to pale yellow colour and a spiky aroma (20, 21). It is naturally produced from the essential oils of plants in the *Lamiaceae, Lauraceae, Myrtaceae* and *Myristicaceae* families (20, 21). It is the most abundant component of clove oil (*Syzygium aromaticum*) (20, 21). Eugenol has been widely used in daily life, for instance, in traditional medicine, dentistry, food mixtures, household supplies and other personal hygiene products (20, 22, 23). It is commonly used as an anticancer agent owing to its potential pharmacological activity (20, 24–27). Previous studies have shown that eugenol induces cell death through the apoptotic pathway in breast cancer, cervical cancer, colon cancer, leukaemia and melanoma cell lines (28–33). The induction of apoptosis by eugenol is characterised by the expression of apoptotic markers and morphological changes in the cells (28–33). Recently, eugenol was reported to suppress malignancy by inhibiting the biological activity of OSCC (34). Several studies have reported the anticancer activity of eugenol and its ability to induce cell death through different pathways in several OSCC cell lines. However, no current review has discussed the extent of eugenol anticancer research on oral cancer cell lines using in vitro studies. Therefore, this systematic review aimed to discuss the anticancer potential of eugenol against oral cancer cell lines in vitro.

## Method

The systematic review was conducted according to the Preferred Reporting Items for Systematic Reviews and Meta-Analysis (PRISMA) guidelines 2020. The protocol for this systematic review was registered in PROSPERO (ID: CRD42023437406) (University of York, York, UK). The study was initiated by identifying the following research questions according to the PICO framework: i) population: oral cancer cell line; ii) intervention: eugenol exposure; iii) comparison: unexposed group (control); and iv) outcome: anticancer effects. This systematic review aimed to answer the following research question: ‘What is the anticancer potential of eugenol against oral cancer cell lines in in vitro studies?’.

### Search Strategy

Search strategies are currently being developed for PubMed, ScienceDirect, Springer Link and EBSCOhost. The keywords used in the databases were ((effect) AND (eugenol) AND ((oral cancer) OR (oral carcinoma) OR (oral malignancy))). Additional literature searches were conducted manually using related keywords to find articles relevant to the research topic. No restrictions were placed on the language or publication date to broadly explore the development of eugenol anticancer research in oral cancer cell lines.

### Inclusion and Exclusion Criteria

The inclusion criteria consisted of articles on in vitro studies available in full text that discuss the anticancer effects of eugenol or its derivative compounds (both synthetic and natural) through various pathways against oral cancer cell lines in vitro. In this study, the term ‘effect’ was defined as the impact of eugenol or its derivative compounds on oral cancer cell lines. This effect may be due to cell death through apoptotic and/or non-apoptotic pathways or other pathways found in the literature review. It may also include the cytotoxicity of eugenol, which inhibits or blocks the growth of oral cancer cell lines. The critical parameters for determining eligible articles included the use of eugenol and its derivatives (regardless of whether they were the primary or comparator compounds), prioritisation of oral cancer cell lines, exclusion of all cell lines other than those derived from oral cancer, and prioritisation of the accessibility and clarity of the article data. The exclusion criteria consisted of literature reviews, in vivo studies, clinical studies, case reports, observational studies, non-experimental studies and gray literature.

### Selection Process

Article identification was conducted by three dependent examiners (SNW, JAA, and WH) from February to March 2023, using keywords inserted into electronic databases. Additional articles were manually searched to identify studies investigating at least one anticancer effect of eugenol or its derivatives on oral cancer cell lines. All documents were organised using Mendeley Dekstop software (Mendeley Ltd., London, UK). Automatic duplication removal was followed by manual rechecking. The articles were initially selected by an examiner (SNW) based on the title and/or abstract, following the specified inclusion and exclusion criteria. Full-text files of the selected articles were downloaded, and those with unavailable full texts were excluded. Subsequently, articles were selected by reading the full text to determine the suitability of each research topic. In cases of uncertainty, the full-text selection was re-evaluated by two reviewers (JAA and WH). Full texts of all potentially eligible studies were obtained, and the inclusion and exclusion criteria were applied again. The final selection of articles was determined through discussion and agreement among the three reviewers (SNW, JAA, and WH), and the selected articles were further assessed for risk of bias before being analysed and synthesised to answer the research questions.

### Studies Risk of Bias Assessment

The quality assessment in this systematic review was conducted by the three dependent reviewers (SNW, JAA and WH) in April 2023 using The Science in Risk Assessment and Policy (SciRAP) tools (Appendix) to evaluate the reliability and relevance of in vitro research according to the three criteria: reporting quality, methodological quality and relevance (35). Reporting quality was assessed based on aspects including test compounds and controls, test systems, dosage and administration, data collection and analysis, funding sources, and competing interests (35). Methodological quality was assessed based on aspects of the research methods, including study design, methods and management, which could affect the final results (35). Relevance was evaluated based on the context of this study. The scores for the three criteria ranged from 0 to 100, with a minimum score of 70 considered good quality (36). Studies scoring above 70 were of good quality with a low risk of bias and were included synthesis analysis. Studies scoring below 70 were of poor quality with a high risk of bias and were excluded. The quality assessment results for each study were compiled and thoroughly compared based on the three criteria.

### Data Extraction and Analysis

Data were extracted and analysed from May 2023 to June 2023. One reviewer (SNW) was initially responsible for collecting data from the included articles. Data extracted from the individual articles included article identity (title, author, year and country of origin), cell line type, eugenol (type, dosage form, source and dose), research method, intervention time, apoptosis markers and conclusion of the anticancer effect. The extracted data were re-evaluated by two independent reviewers (JAA and WH) and presented in tabular form. After reaching an agreement among the three reviewers, analysis and synthesis were performed. In a systematic review, analysis synthesis refers to the process of combining and evaluating the findings of individual studies to determine the review outcomes (37–39). Meta-synthesis was applied as the analysis synthesis method by summarising and reviewing the research results in each article using a descriptive qualitative approach (37–39). The reviewers (SNW, JAA and WH), then analysed and synthesised the collected data through discussion to achieve a mutually acceptable outcome. Data integration was performed to obtain a deeper understanding of the anticancer effects of eugenol on oral cancer cell lines based on the results of previous in vitro studies.

## Results

### Study Selection Results

**Figure 1:**
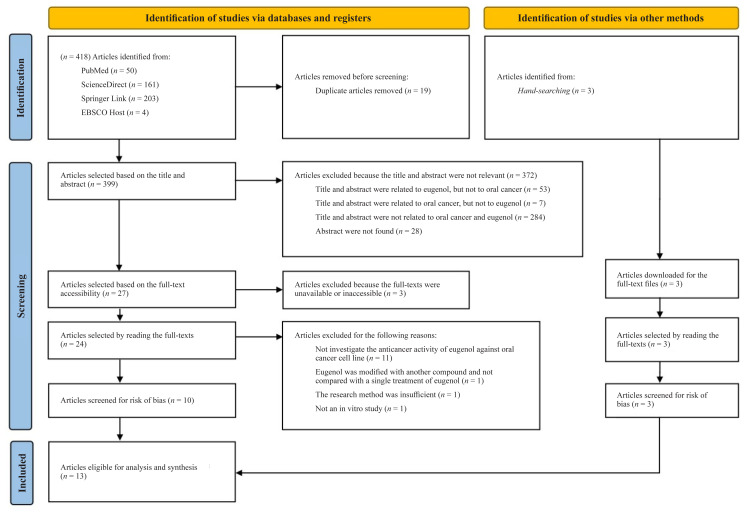
PRISMA 2020 flow diagram

The selection process identified 418 articles (50 articles from PubMed; 161 articles from ScienceDirect; 203 articles from SpringerLink and 4 articles from EBSCOhost). After removing 19 duplicate articles, a total of 399 articles remained. Initial screening of titles and abstracts resulted in the selection of 27 articles, while 372 articles were excluded for the following reasons: i) 53 articles were associated with eugenol but not oral cancer; ii) 7 articles were associated with oral cancer but not eugenol; iii) 284 articles were not associated with either oral cancer or eugenol; and iv) abstracts of 28 articles were not found. Full-text files of the obtained articles were downloaded, leaving 24 articles after excluding 3 articles for which full text was unavailable. The second screening involved reading the full text to determine the suitability of the research topic, resulting in 10 articles for further analysis. Fourteen articles were excluded for the following reasons: i) 11 articles did not investigate the anticancer activity of eugenol against oral cancer cell lines; ii) one article used eugenol modified with another compound and did not compare it with a single treatment of eugenol; iii) one article did not sufficiently explain the research method; and iv) one article was not an in vitro study. Additionally, manual searches were performed, yielding three more articles relevant to the topic. Thus, 13 articles were included in the final analysis and synthesis. The flow of the literature search based on the PRISMA guidelines 2020 is shown in Figure 1.

### Characteristic of the Included Studies

Table 1 shows the main characteristics of the reviewed articles, including author, country, year of publication, cell lines used, type of eugenol, dosage form, source of eugenol, research methods, effective dose/concentration, intervention time, apoptosis markers and the conclusion of the anticancer effect of eugenol on oral cancer cell lines. Most articles were from Asian countries, with one article from India (40), eight from Japan (41–48), one from Taiwan (49) and two from Korea (50, 51). Another study was conducted in Romania (52). All the included articles had an in vitro study design.

According to the findings of these studies, the oral cancer cell lines used vary widely, including SCC-4 (52), SCC-25 (40, 51), HSC-2 (41, 44, 50), HSC-4 (41), Ca9-22 (41), HSG (42, 43, 45–48) and OC-2 (49). Most of these studies used synthetic eugenol as the test compound (*n* = 11) (41–48, 50–52), whereas others used natural eugenol derived from plant extracts (*n* = 1) (40) or a zinc oxide eugenol-based root canal sealer (*n* = 1) (49). The research methods, eugenol doses/concentrations and intervention times were heterogeneous. Three articles reported the expression of apoptotic markers, indicating apoptotic cell death caused by eugenol treatment (50–52).

### Risk of Bias in the Included Studies

A summary of the quality assessment of the studies using SciRAP tools is shown in Tables 2 and 3. The highest reporting quality score of 84.78 was obtained in the study of Fujisawa et al. (46), while the lowest score of 73.91 was obtained in studies by Koh et al. (41) and Sohn et al. (51). The highest methodological quality score of 85.71 was obtained by Huang et al. (49), whereas the lowest score of 71.43 was recorded by Sohn et al. (51). All included articles scored above the minimum score limit were of good quality. The relevance assessment indicated that all articles were directly relevant, with an overall low risk of bias.

## Discussion

Eugenol is a phenolic aromatic compound with the IUPAC name 2-methoxy-4-(2-propenyl) phenol (20). It is consistently oily, transparent to pale yellow in colour and has a spicy-like aroma (20). Owing to its anticancer properties, eugenol can act as a chemopreventive agent. This systematic review discusses the potential anticancer effects of eugenol in the treatment of oral cancers. The selected articles provided evidence for the ability of eugenol to induce cell death through apoptotic and non-apoptotic pathways, inhibit cell proliferation and affect oxidative stress, thereby contributing to cell death in several oral cancer cell lines in in vitro studies.

### Eugenol Induces Apoptosis and Inhibits Proliferation of OSCC Cell Line

Eugenol has potential as an anticancer agent in OSCC cell lines. The research findings of Surducan et al. (52), Varadarajan et al. (40) and, Kim and Park (50) demonstrated several anticancer activities of eugenol, indicating its potential use as an anticancer treatment. These studies reported that eugenol is cytotoxic, induces apoptosis through specific molecular pathways, and inhibits the growth and survival of the OSCC cell lines SCC-4, SCC-25 and HSC-2 (40, 50, 52).

The apoptotic pathway is a beneficial mechanism in anticancer therapy that regulates the uncontrolled growth of cancer cells. Apoptosis is the natural mechanism of cell death following exposure to certain stimuli (53). Under physiological conditions, apoptosis is also known as programmed cell death (54). Apoptotic cells undergo several morphological changes, including chromatin condensation and nuclear fragmentation, which are accompanied by increasingly rounded cells, cell volume reduction (pyknosis) and pseudopod retraction (53, 54). In the late stage of apoptosis, visible changes in cell morphology include the loss of cell membrane integrity, membrane blebbing and alteration of the ultrastructure of cytoplasmic organelles (53, 54). According to Surducan et al. (52), Varadarajan et al. (40) and Kim and Park (50), eugenol treatment induces OSCC cell death via an apoptotic mechanism. OSCC cell morphology showed signs of apoptotic cells after eugenol treatment. Morphology evaluation conducted under fluorescent microscopy by Surducan et al. (52) resulted in images of rounded and floating cells, loss of attachment with the surrounding cells and dose-dependent reduction in confluence. In DAPI and phalloidin staining, the formation of apoptotic bodies, condensation and fragmentation of chromatin, and condensation of actin filaments in the nucleus were observed (52). The condensed nuclei appeared rounded and shrunken, and fluorescence was more intense under a microscope (52). This study was supported by Kim and Park (50), who used DAPI staining to confirm morphological changes in the nucleus. The results showed that the nucleus was destroyed by DNA fragmentation, followed by an increase in the proportion of apoptotic bodies in a dose-dependent manner (50). When compared to previous research by Vidhya and Devaraj (50), the effects of eugenol on cancer cell lines were similar (29). A previous study reported that eugenol dose-dependently increased apoptotic cells and DNA fragmentation in the breast cancer cell line MCF-7 (29). Meanwhile, in acridine orange/ethidium bromide staining by Varadarajan et al. (40), cells with eugenol treatment at a 25 μM concentration appeared yellowish, illustrating the early phase of apoptosis, and orange, illustrating the late phase of apoptosis. However, the findings of Varadarajan et al. (40) differed from those of Das et al. (28). In the study by Das et al. (28), eugenol induced early apoptosis in HeLa cells at 0.5 mg/mL dose, indicated by a greenish-yellow colour. However, at 1 mg/mL, eugenol increased the number of necrotic cells, as indicated by a reddish orange colour accompanied by the disintegration of apoptotic cells (28). Based on cell morphology analysis, it was found that the HeLa cervical cancer cell line exhibited apo-neurosis features consisting of nucleus condensation after being exposed to eugenol at a concentration of 0.5 mg/mL–1 mg/mL for 24 h (28).

After evaluating the changes in cell morphology, the molecular mechanisms underlying the ability of eugenol to induce apoptosis through the expression of pro-apoptotic and anti-apoptotic genes were investigated. There are two pathways for apoptosis: the intrinsic pathway, involving mitochondria through the initiation of caspase-9 and the subsequent activation of caspases 3/6/7, and the extrinsic pathway, involving Fas ligand/FASL or tumour necrosis factor (53, 54). In this systematic review, we found that eugenol induced apoptosis in OSCC cell lines through an intrinsic pathway. The intrinsic apoptotic pathway depends on the regulation of the Bcl-2 family (52–54). The Bcl-2 family of proteins is comprised of two major subgroups: anti-apoptotic proteins (Bcl-2 and Bcl-XL) and pro-apoptotic proteins (Bax and Bak) (53, 54). Caspase-3 is the main factor involved in apoptosis induction (52). Caspase-3 is usually inactive in the nucleus and mitochondrial outer membrane (52). Caspase-3 is activated when it receives stimulation that induces apoptosis, such as the release of cytochrome c into the cytoplasm (52, 54). Activation of pro-apoptotic genes leads to the permeabilisation of the mitochondrial outer membrane, releasing cytochrome c into the cytoplasm (53, 54). Cytochrome c combines with apoptotic protease-activating factor-1 (APAF-1), dATP and procaspase-9 to form apoptosomes (53, 54). Apoptosomes convert procaspase-9 into caspase-9, which activates caspase-3 and caspase-7 (54). These two caspases damage cellular proteins, leading to death (54). Based on the findings of this systematic review, eugenol increased the expression of the pro-apoptotic genes Bax, Bak and Bad, as well as the production of cleaved caspase-3 in several OSCC cell lines (34, 50, 52). The pro-apoptotic genes Bax and Bad were found to be expressed significantly more in SCC-4 cells, according to Surducan et al. (52). However, the expression of the anti-apoptotic gene Bcl-2 was not significantly affected (52). Similar results were reported by Duan et al. (34), who noted an increase in the expression of cleaved poly-ADP ribose polymerase (PARP), cleaved caspase-3 and the pro-apoptotic gene Bax in SCC-9 cells. In the present study, eugenol decreased the expression of the antiapoptotic gene Bcl-2 (34). This finding was supported by Kim and Park (50), who observed changes in cleaved caspase-3, which increased after eugenol treatment in HSC-2 cells. Kim and Park (50) showed that the expression of the pro-apoptotic gene Bak increased significantly, whereas that of the anti-apoptotic gene Bcl-xl was not substantially affected. These results are consistent with those of earlier studies on the influence of eugenol on breast cancer, cervical cancer, colon cancer and leukaemia (28, 30–32). Jaganathan et al. (31), who investigated the effects of eugenol on the colon cancer cell lines HCT-15 and HT-29, reported that cleaved caspase-3 and cleaved PARP levels were increased. Similar findings were also reported by Das et al. (28) and Al-Sharif et al. (30), who showed an increase in the expression of the pro-apoptotic gene Bax, cleaved caspase-3, and cleaved PARP in the cervical cancer cell line HeLa and several breast cancer cell lines. The molecular mechanism of eugenol-induced apoptosis was supported by Yoo et al. (32), who studied the promyeloid leukaemia cell line, HL-60. The study reported a comprehensive anticancer mechanism of eugenol against cancer cell lines, involving the promotion of translocation of the pro-apoptotic gene Bax, a decrease in the anti-apoptotic gene Bcl-2, the release of cytochrome c, and the activation of caspase-9 and caspase-3.

Eugenol acts synergistically with other compounds to enhance its anticancer effects in OSCC cell lines. Sohn et al. (51) examined the apoptotic effects of cotreatment with eugenol and Chios Gum Mastic (CGM) in SCC-25 cells, observing a decrease in the anti-apoptotic gene Bcl-2 and an increase in the pro-apoptotic gene Bax (51). The present study also revealed several apoptotic signs in cells, including nuclear condensation, DNA fragmentation, release of cytochrome c into the cytosol, translocation of AIF and DFF40 (CAD) into the nucleus, and activation of caspase-3, caspase-6, caspase-7, caspase-9, PARP, Lamin A/C and DFF45 (ICAD) (51). Similarly, Varadarajan et al. (40) reported the synergistic effects of eugenol with other compounds in *Cinnamomum verum* extract to enhance the anticancer effect on SCC-25 cells. This study reported the synergistic effects of eugenol with active compounds, including cinnamaldehyde and 4-hydroxycinnamic acid, as well as polyphenols, tannins and saponins (40). Intervention with *C. verum* extract led to cells dominated by a reddish orange coloured late apoptotic phase, a ladder pattern DNA picture indicating apoptosis, and significant loss of mitochondrial membrane potential (40). Further research is required on its interaction with other treatment modalities to determine its potential as an anticancer therapy for managing OSCC.

However, Sohn et al. (51) reported that the results of a single treatment with eugenol did not show significant results compared to the co-treatment of eugenol with CGM. A single treatment with eugenol (0.5 mM) for 24 h slightly reduced cell viability and DNA fragmentation, with only a few cells with hypoploid DNA undergoing apoptosis (51). In the SCC-25 cell line treated with a single eugenol molecule, caspase-6 was activated, accompanied by a decrease in Bcl-2 and an increase in Bax (51). In contrast, Varadarajan et al. (40) found that a single treatment of eugenol with a concentration of 25 μM for 48 h induced apoptosis, suggesting that the induction of apoptosis depends on the type of eugenol used and the duration of eugenol treatment in OSCC cell lines.

Eugenol also inhibited the growth and survival of OSCC cell lines through specific cell cycle changes (34, 40). Varadarajan et al. (40) reported a dose-dependent increase in the cell population in the S phase of the SCC-25 cell line. Eugenol plays an important role in inhibiting cancer cell proliferation by arresting cells in the S phase and increasing the corresponding sub-G0 population (40). Similar results were reported by Choi et al. (33) for the melanoma cell line G361, where eugenol increased the number of cells in the S phase and decreased the number of cells in G1 and G2/M phases (33). This led to an increase in the number of cells undergoing apoptosis in the G361 melanoma cell line, indicating that eugenol caused cell cycle arrest in the S phase before apoptosis begins to develop (33). Kim and Park (50) discovered a considerable increase in the ratio of cells in the sub-G1 phase as the concentration increased in HSC-2 cell line. During this phase, eugenol induces cell shrinkage, indicating apoptosis in the cell cycle (50). Our findings are consistent with those of Jaganathan et al. (31) and Júnior et al. (55), who reported the apoptotic effect of eugenol on the colon cancer cell lines HCT 15 and HT 29, breast cancer cell lines MDA-MB-231 and MCF-7, cervical cancer cell line SIHA, and melanoma cell lines SK-Mel-28 and A2058. In HCT-15 and HT-29 cell lines, eugenol significantly increased cell accumulation in the sub-G1 phase in a time-dependent manner, whereas in other cell lines, eugenol suppressed the G2/M phase and caused DNA damage, leading to apoptosis through the emergence of the sub-G0/G1 phase (31, 55). These findings support the antiproliferative properties of eugenol and its ability to induce apoptosis in cancer cell lines.

A recent study reported that eugenol inhibited the growth and survival of OSCC cell lines by targeting macrophage migration inhibitory factors (MIF), which plays a role in cancer progression (34). MIF is a pleiotropic cytokine that is pro-inflammatory and involved in tumorigenesis (56, 57). MIF is considered a pro-tumour factor because of its ability to support tumour progression. According to previous studies, MIF is a novel prognostic marker for patients with OSCC (56). Upregulation of MIF expression has been observed in OSCC cell lines and can trigger proliferation, migration, and invasion (56, 57). Eugenol can bind to MIF, leading to decreased MIF expression in OSCC cell lines (34). By targeting MIF, eugenol has the potential to inhibit key cellular processes in cancer cells that contribute to cancer cell growth. Previous research has also shown that matrix metalloproteinases (MMP)-2 and MMP-9 are required by OSCC cell lines to stimulate proliferation, migration, and invasion, which results in the upregulation of MIF in OSCC cell lines and increased MMP-2 and MMP-9 expression (57). This finding is consistent with a study by Duan et al. (34), who reported that eugenol decreased cell proliferation and reduced the ability to invade and migrate, along with decreased expression of MMP-2 and MMP-9 in SCC-9 cell lines.

### Eugenol Induces Oral Cancer Cell Death through Non-Apoptotic Pathways

Based on these findings, eugenol can induce cell death via a non-apoptotic pathway. In contrast to the studies by Surducan et al. (52), Varadarajan et al. (40), and Kim and Park (50), which showed apoptosis induction in OSCC cell lines by eugenol, Koh et al. (41) reported that the OSCC cell lines HSC-2, HSC-4 and Ca9-22 exhibited cell death without the release of apoptotic markers or DNA fragmentation. Activation of caspase-3 and caspase-7 only occurred in eugenol treatment for 6 h–24 h and was not observed in treatments for less than 6 h. In addition to caspase-dependent apoptosis, necroptosis, a type of necrotic cell death controlled by receptor-interacting protein 1 (RIP1), RIP3 and mixed lineage kinase domain-like, is another form of programmed cell death (58). Necroptosis is a fail-safe mechanism to eliminate cells that respond to stress owing to the failure to undergo apoptosis (58). Necroptosis is characterised by both necrosis and apoptosis. The morphology of necroptotic cells that characterises necrosis includes a transparent cytosol, early loss of plasma membrane integrity and enlarged mitochondria (58, 59). The morphology of necroptotic cells that characterises apoptosis includes condensation and fragmentation of the nucleus and other cellular organelles, plasma membrane blebbing and cell shrinkage (58, 59). However, further research is necessary to determine the cell death pathways in OSCC cell lines based on morphological characteristics and markers of apoptosis and necroptosis. Multiple factors, including cell type, genetics, stimuli, intracellular redox, pH and ion levels, determine the selection of the cell death pathway (58).

Similar results were observed in eugenol-treated HSG cells. Atsumi et al. (42) stated that eugenol, bis-eugenol, isoeugenol, and α-diisoeugenol with a cytotoxic concentration of 50% could not induce apoptosis in HSG cell line. Additionally, Atsumi et al. (43) discovered that after receiving treatment with α-diisoeugenol, the number of cells in the early apoptotic phase was equivalent to the control. However, at a concentration of 15 μM, the presence of late apoptosis phase cells or necrosis cells was seen to increase rapidly (43). This is similar to the results of Das et al. (28), who found an increase in necrotic cells accompanied by the disintegration of apoptotic cells in a HeLa cervical cancer cell line treated with eugenol at a dose of 1 mg/mL. Necrotic cells are morphologically characterised by the swelling of organelles, early rupture of plasma membranes, and release of cellular materials into tissues, triggering the inflammatory process (60). Therefore, necrosis is considered a more harmful cell death pathway than apoptosis (60).

### Relationship of Cytotoxicity and Oxidative Stress of Eugenol on Oral Cancer Cell Line

Koh et al. (44) investigated the metabolic changes induced by eugenol in OSCC cell line HSC-2. These results reveal a potential mechanism of action underlying the anticancer effect of eugenol and explain the changes in cell metabolism resulting from eugenol treatment (44). At cytotoxic doses, eugenol causes a reduction in ATP utilisation, with a noticeable decrease in the ratios of AMP to ATP and ADP to ATP (44). Reduced ATP utilisation can interrupt cellular processes that require ATP, thereby triggering necrosis (44). During necrosis, mitochondrial dysfunction occurs due to the prolonged opening of the mitochondrial permeability transition pore (MPTP) (60). This promotes the formation of reactive oxygen species (ROS), loss of ATP synthesis, organelle enlargement and depolarisation of the mitochondrial inner membrane (60). Koh et al. (44) reported that eugenol induces increased levels of polyamines and glycolytic metabolites. An increase in polyamines is a cellular response to repair membrane damage, as eugenol may induce damage to the lipid layer of the cell membrane (44). Eugenol induces oxidative stress in HSC-2 cells, which is characterised by increased levels of the oxidised forms of glutathione, cysteine-glutathione disulphide and methionine sulfoxide. Eugenol has been reported to reduce glutathione and increase lipid peroxidation products in the breast cancer cell line MCF-7 (29). Based on these findings, the sequence of intracellular processes after eugenol intervention begins with the induction of oxidative stress, followed by cell membrane damage accompanied by repair and ends with a decrease in ATP utilisation (44).

A previous study by Atsumi et al. (45) showed that the cytotoxicity of eugenol with visible light (VL) irradiation on the HSG cell line was caused by the formation of eugenol radicals and ROS. Atsumi et al. (45) also investigated the photocytotoxicity of eugenol in solutions at different pH levels. The production of radicals and ROS increased with higher eugenol doses, longer irradiation times and higher pH levels. Glutathione and cysteine protect cells from damage caused by eugenol under visible light irradiation (45). The results of this study were strengthened by Fujisawa et al. (46), who showed that eugenol produced free radicals at pH 9.5 and induced oxidative stress in HSG cells. The pH-dependent nature of ROS suggests that the ability of eugenol to induce cytotoxicity varies under various physiological conditions. This finding emphasises the importance of considering pH levels when developing eugenol for therapeutic applications. According to Fujisawa et al. (46), free radicals produced by eugenol can be removed by adding 2-ethoxy benzoic acid (EBA), which reduces the cytotoxicity of eugenol. Acetylsalicylic acid increases the intensity of eugenol radicals at low concentrations but eliminates them at high concentrations (46). However, these results contradict those reported by Atsumi et al. (42, 43) and Fujisawa et al. (47). Eugenol induced cytotoxicity in HSG cell lines but did not induce ROS production compared to curcumin (42, 43, 47). Similarly, a-diisoeugenol, isoeugenol and bis-eugenol compounds did not induce ROS despite being cytotoxic to HSG cell lines (42, 43, 47).

### Cytotoxic and Genotoxic of Eugenol in Dental Products on Oral Cancer Cell Line

According to previous reports, eugenol exerts cytotoxic effects on several oral cancer cell lines. Previous studies have reported the cytotoxicity of eugenol, a compound commonly used in dental products, against the OSCC cell lines HSC-2, HSC-4, and Ca9-22 (41). It is also cytotoxic to SCC-4 and SCC-25 cells and induces apoptosis (52). According to Atsumi et al. (42), α-diisoeugenol showed the most cytotoxicity against the HSG cell line compared to other compounds. The order of cytotoxicity from the highest to the lowest was α-diisoeugenol > isoeugenol > bis-eugenol > eugenol (42). However, these results contradict the findings of Fujisawa et al. (48), who explored the application of bis-eugenol in zinc oxide eugenol cement. Eugenol was found to be more cytotoxic than bis-eugenol to HSG cells (48). This contradiction may have occurred because the eugenol used in the two studies was obtained from different sources and was influenced by the dose/concentration of eugenol and the cell line incubation time.

Koh et al. (41) compared the cytotoxicity of eugenol against OSCC and normal oral cell lines (human gingival fibroblasts, human pulp cells and human periodontal ligament fibroblasts). Eugenol showed minimal cytotoxic effects on normal oral cell lines. A previous study reported minimal cytotoxicity of a zinc-oxide eugenol-based root canal sealer (Endofill) on macrophage cells compared to a non-zinc-oxide eugenol-based root canal sealer (Sealer 26) (61). Eugenol is cytotoxic to human dental pulp cells and induces the expression of molecular markers of osteogenic differentiation (62). This suggests that eugenol does not selectively target cancer cells. Hence, the use of eugenol for topical application in dental products should be carefully monitored. Nonetheless, the cytotoxicity of eugenol can be reduced by mixing it with other compounds that can minimise its cytotoxicity. Fujisawa et al. (46) reported that eugenol has a lower cytotoxicity than 2-ethoxybenzoic acid (EBA) and acetylsalicylic acid at low concentrations; however, its cytotoxic activity increases rapidly above the micelle formation concentration. By adding EBA, the cytotoxicity of eugenol can be reduced because EBA eliminates the production of free radicals (47). This suggests that the addition of EBA improves the development of safer and more effective dental products. This study also provided information on the appropriate dose and safe concentration of EBA, which may be beneficial for developing better and more efficient dental products (46). Fujisawa et al. (48) reported similar results. This study investigated the mechanical and antimicrobial properties of bis-eugenol and its potential to improve the properties of zinc oxide eugenol (48). The addition of bis-eugenol did not reduce the physical properties of zinc oxide eugenol and was less toxic than eugenol (48). Thus, bis-eugenol, especially zinc oxide-eugenol, may also be applicable to the development of dental products.

Huang et al. (49) evaluated the genotoxicity of zinc oxide eugenol-based root canal sealers. This study compared the genotoxicity of zinc oxide eugenol-based root canal sealers (Canals and Tubilseal) with that of other sealers, including calcium hydroxide- and epoxy resin-based sealers. The results showed that zinc oxide eugenol-based root canal sealers did not produce significant genotoxic effects compared with other sealers (49). Canals and Tubilseal cause a dose-dependent increase in DNA damage in the OC2 cell line (49). However, the increased shape factor and migration in OC2 cell lines after treatment with Canals and Tubilseal were not always dose-dependent but occasionally reached a maximum at mid-dose (49). A more recent study reported that eugenol caused DNA damage at higher concentrations and protected human lymphocytes from oxidative DNA damage induced by H_2_O_2_ (63). Eugenol did not show genotoxic effects at concentrations below the IC50 value but could potentially cause DNA damage at higher concentrations (63). In contrast, Han et al. (64) reported the potential of eugenol as a chemopreventive agent against the genotoxicity induced by 7,12-dimethylbenz[a]anthracene (DMBA) in the breast cancer cell line MCF-7. Eugenol inhibits DMBA-DNA formation by decreasing the expression of CYP1A1 and CYP1B1, which play a role in DMBA metabolism and increasing the expression of NAD(P)H: quinone oxidoreductase (QR), the main detoxification enzyme for DMBA (64). Eugenol had a bifunctional effect on CYP1 and QR and is thus an effective protective agent against DMBA-induced genotoxicity (63). These findings suggest that eugenol acts as a chemopreventive agent against DMBA-induced carcinogenesis (64). Therefore, eugenol may potentially be utilised as a chemopreventive agent in OSCC cell lines; however, it should be used with caution at higher concentrations. Further in vivo investigations and clinical trials are required to establish the genotoxic or antigenotoxic potential of eugenol and to validate its effectiveness and safety as a therapeutic agent for the management of OSCC.

### Limitations and Suggestions

There were limitations in accessing full-text documents, which made it impossible to include gray literature and other articles without full text despite their relevance to the research topic. Additionally, the data in the included articles were heterogeneous regarding the type of cell line used, the dose of eugenol applied and the research methods employed. This heterogeneity made it difficult to compare the effects of eugenol on oral cancer cell lines. Therefore, further research is required to directly and simultaneously compare the anticancer effects of eugenol in several oral cancer cell lines using specific methods.

## Conclusion

Eugenol is a potential anticancer agent for OSCC. It induces cell death through apoptotic and non-apoptotic pathways, inhibits cell proliferation and affects oxidative stress, contributing to cell death in several oral cancer cell lines.

## Figures and Tables

**Table 1:** Main characteristics of the reviewed articles

No.	Author (country, year)	Cell line	Eugenol (types)	Dosage form (source)	Methods	Dose/Concentration	Time	Apoptosis marker	Anticancer effect conclusion
1	Surducan et al. (Romania, 2023) (52)	SCC-4	Eugenol (synthetic)	Solution, liquid (Sigma Aldrich, Germany)	MTT assay	1 mM	72 h	Bax	Eugenol showed a cytotoxic effect on SCC-4 cell line by inducing apoptosis. The morphological changes of apoptotic cells were observed, accompanied by an increase in apoptotic markers.
					Morphology evaluation	1 mM	72 h	Bad	
					Nuclear and cytoskeletal evaluation	0.5 mM	72 h		
					RT-PCR	0.5 mM	72 h		
2	Varadarajan et al. (India, 2020) (40)	SCC-25	Eugenol (natural)	Ethanolic extract Hydroalcoholic extract Aqueous extract (*Cinnamomum verum*)	MTT assay	24.71 μM	48 h	(Not measured)	Eugenol exerted anticancer effects on SCC-25 cell line by inducing apoptosis and arresting S phase. The apoptosis induction occurred through the alteration of mitochondrial membrane potential.
					AO/EB staining	25 μM	48 h		
					DNA fragmentation assay	25 μM	48 h		
					Flow cytometry	25 μM	48 h		
					Determination of mitochondrial membrane potential	25 μM			
3	Koh et al. (Japan, 2013) (41)	HSC-2	Eugenol (synthetic)	Solution, liquid (Wako Pure Chemical, Osaka, Japan)	MTT assay	732 μM	24 h	(Not measured)	Apoptosis was not involved in the growth inhibition of OSCC cell line at an early stage induced by eugenol. Cell death occurred without the induction of apoptotic markers.
		HSC-4				734 μM	24 h		
		Ca9-22				750 μM	24 h		
					Western blotting assay	1464 μM	24 h		
						1468 μM	24 h		
						1500 μM	24 h		
4	Koh et al. (Japan, 2013) (44)	HSC-2	Eugenol (synthetic)	Solution, liquid (Wako Pure Chemical, Osaka, Japan)	CE-TOF-MS analysis	2.8 mM	4 h	(Not measured)	Metabolic changes in eugenol-mediated OSCC cell line showed induction of non-apoptotic cell death. Cytotoxic concentrations of eugenol induced a decrease in ATP utilisation, oxidative stress, and an increase in polyamines and glycolytic metabolites.
5	Atsumi et al. (Japan, 2006) (42)	HSG	Bis-eugenol Eugenol α-diisoeugenol Isoeugenol (synthetic)	Solution, liquid (synthesis result)	MTT assay	0.182 μM	24 h	(Not measured)	Regarding cytotoxicity, α-diisoeugenol was the most cytotoxic, followed by isoeugenol, bis-eugenol, and eugenol. However, these compounds did not induce apoptosis or ROS in HSG cell lines.
						0.287 μM	24 h		
						0.003 μM	24 h		
						0.059 μM	24 h		
6	Atsumi et al. (Japan, 2005) (43)	HSG	α-diisoeugenol (synthetic)	Solution, liquid (synthesis result)	MTT assay	0.0027 mM	24 h	(Not measured)	Neither apoptosis nor ROS was induced despite α-diisoeugenol being cytotoxic to HSG cells.
					Annexin V-FITC/PI assay	10 μM	4 h		
					Detection of ROS	1000 μM	30 min		
7	Fujisawa et al. (Japan, 2003) (46)	HSG	Eugenol (synthetic)	Solution, liquid (Tokyo Kasei Chem. Co., Tokyo, Japan)	MTT assay	0.52 mM	24 h 1 min–20 min	(Not measured)	Eugenol produced free radicals at pH 9.5 and caused oxidative stress in HSG cell line. The cytotoxicity of eugenol was lower than 2-ethoxybenzoic acid and acetylsalicylic acid at low concentrations but increased rapidly above the micelle-formation concentration.
					ESR electroscopy	100 mM			
8	Huang et al. (Taiwan, 2001) (49)	OC2	Canals tubilseal (root canal sealers)	Pasta (Showa Corporation, Tokyo, Japan and Kerr)	Single-cell gel	0.5 mg/mL	24 h	(Not measured)	Root canal sealers containing eugenol could be genotoxic and induce a dose-dependent increase in DNA damage.
					electrophoresis assay (comet assay)	2.5 mg/ml	24 h		
9	Atsumi et al. (Japan, 2001) (45)	HSG	Eugenol (synthetic)	Solution, liquid (Tokyo Kasei Chem. Co., Tokyo, Japan)	Cytotoxicity assay			(Not measured)	The cytotoxicity of visible light irradiated eugenol was due to the formation of eugenol radicals and ROS, which were produced depending on eugenol dose, irradiation time, and medium pH.
					No VL irradiation	2.3 × 10 M^−4^	30 min		
					5 min VL irradiation	1.0 × 10 M^−4^	30 min		
					10 min VL irradiation	7.0 × 10 M^−5^	30 min		
					Detection of ROS				
					No VL irradiation	1 mM	30 min		
					5 min VL irradiation	1 mM	30 min		
					10 min VL irradiation	1 mM	30 min		
10	Fujisawa et al. (Japan, 1999) (48)	HSG	Eugenol Bis-eugenol (synthetic)	Solution, liquid (Tokyo Kasei Chem. Co., Tokyo, Japan and synthesised)	MTT assay	10 M^−3^	48 h	(Not measured)	Eugenol was found to be more cytotoxic than bis-eugenol in HSG cell line.
						10 M^−3^	48 h		
11	Kim et al. (Korea, 2015) (50)	HSC-2	Eugenol (synthetic)	Solution, liquid (Source not mentioned)	MTT assay	2 mM	24 h	Cleaved	Eugenol caused morphological changes in the cell nucleus, with the ratio of apoptotic bodies increasing in a concentration-dependent manner. The cell nucleus was destroyed due to DNA fragmentation, and several apoptotic markers that characterise apoptosis were found.
					DAPI dying	2 mM	24 h	Caspase3	
					Flow cytometric	2 mM	24 h	Bak	
					Electrophoretic protein	2 mM	–	Bcl-xl	
12	Sohn et al. (Korea, 2011) (51)	SCC-25	Eugenol (synthetic)	Solution, liquid (Sigma, St. Louis, MO, USA)	MTT assay	0.5 mM	24 h	Bcl-2	The viability reduction and apoptosis induction of SCC-25 cell line by eugenol at a concentration of 0.5 mM for 24 h was not significant. SCC-25 cell line underwent significant apoptosis in the co-treatment of eugenol with Chios Gum Mastic compared to the single treatment of eugenol.
					Hoechst staining	0.5 mM	24 h	Bax	
					TUNEL technique	0.5 mM	24 h	Caspase6	
					Western blot analysis	0.5 mM	24 h		
					Immunofluorescent staining	0.5 mM	24 h		
					Flow cytometry analysis	0.5 mM	24 h		
13	Fujisawa et al. (Japan, 2004) (47)	HSG	Eugenol Isoeugenol Bis-eugenol (synthetic)	Solution, liquid (Tokyo Kasei Chem. Co., Tokyo, Japan and synthesised)	MTT assay	~0.2 mM	24 h	(Not measured)	Eugenol, bis-eugenol, and iso-eugenol were cytotoxic to HSG cell line but did not induce ROS production.
						~0.03 mM	24 h		
						~0.1 mM	24 h		
					Detection of ROS	1 mM	30 min		
						1 mM	30 min		
						1 mM	30 min		

**Table 2:** Reporting quality assessment of the included studies

No.	SciRAP criterion	Reporting quality																							Score

		Test compound and controls							Test system					Administration of test compound				Data collection and analysis					Funding and competing interest		

		1	2	3	4	5	6	7	8	9	10	11	12	13	14	15	16	17	18	19	20	21	22	23	
1.	Surducan et al. (2023) (52)																								82.61
			
2.	Varadarajan et al. (2020) (40)																								78.26
			
3.	Koh et al. (2013) (41)																								73.91
			
4.	Koh et al. (2013) (44)																								82.61
			
5.	Atsumi et al. (2006) (42)																								80.43
			
6.	Atsumi et al. (2005) (43)																								76.09
			
7.	Fujisawa et al. (2003) (46)																								84.78
			
8.	Huang et al. (2001) (49)																								78.26
			
9.	Atsumi et al. (2001) (45)																								82.61
			
10.	Fujisawa et al. (1999) (48)																								80.43
			
11.	Kim et al. (2015) (50)																								76.09
			
12.	Sohn et al. (2011) (51)																								73.91
			
13.	Fujisawa et al. (2004) (47)																								82.61

Notes: 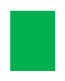 = fulfilled 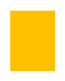 = partially fulfilled 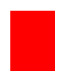 = not fulfilled 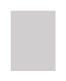 = not determined

**Table 3:** Methodological quality assessment of the included studies

No.	SciRap criterion	Methodological quality															Score

		Test compound and controls					Test system		Administration of test compound			Data collection and analysis					

		1	2	3	4	5	6	7	8	9	10	11	12	13	14	15	
1.	Surducan et al. (2023) (52)																78.57
			
2.	Varadarajan et al. (2020) (40)																78.57
			
3.	Koh et al. (2013) (41)																82.14
			
4.	Koh et al. (2013) (44)																78.57
			
5.	Atsumi et al. (2006) (42)																82.14
			
6.	Atsumi et al. (2005) (43)																82.14
			
7.	Fujisawa et al. (2003) (46)																75.00
			
8.	Huang et al. (2001) (49)																85.71
			
9.	Atsumi et al. (2001) (45)																82.14
			
10.	Fujisawa et al. (1999) (48)																75.00
			
11.	Kim et al. (2015) (50)																78.57
			
12.	Sohn et al. (2011) (51)																71.43
			
13.	Fujisawa et al. (2004) (47)																78.57

Notes: 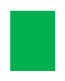 = fulfilled 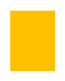 = partially fulfilled 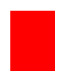 = not fulfilled 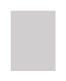 = not determined 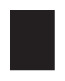 = not applicable

## Appendix

### SciRAP Reporting Quality Criteria per Evaluation Domain

#### Test compound and controls

1. The chemical name or other identification, such as CAS-number, of the test compound was given
2. The purity of the test compound was stated or is traceable according to information given regarding manufacturer and lot/batch number. In case of mixtures, the composition of different constituents was stated
3. The solubility of the test compound was described
4. The solvent (vehicle) was described
5. It was stated that a solvent (vehicle) control was included

#### Test system

6. The test system (e.g., cell line/cells/tissue/organ/embryo/sub-cellular fractions) was described
7. The source of the test system was stated
8. The metabolic competence, i.e., competence of the test system to metabolize the test compound into an active metabolite was described
9. The number of cell passages of the cell line used, was stated. (Remove this criterion if the study was not conducted in a cell line.)
10. Composition of media was described, including use of serum, antibioticsetc.
11. Incubation temperature, humidity, and CO2 concentration were described
12. Measures taken for avoiding or screening for contamination by mycoplasma, bacteria, fungi and virus were described

#### Administration of test compound

13. The administered dose levels or concentrations were stated
14. Cell density or number of cells used during treatment was described. (Remove this criterion if the study was not conducted in a cell line.)
15. The duration of treatment was stated
16. The number of replicates per dose level/concentration or the number of times the experiment was repeated was stated

#### Data collection and analysis

17. The tests and/or analytical methods used were sufficiently described to allow for evaluation of reliability of results
18. The time points for data collection were stated
19. It was stated that the effect of the test compound on cytotoxicity was measured
20. All results were clearly presented
21. The statistical methods and software used were described

#### Funding and competing interests

22. The funding sources for the study were stated
23. Any competing interests were disclosed or it was explicitly stated that the authors did not have any competing interests

#### Other

24. Was all information that is indispensable for evaluating the reliability of data given? This includes information on the test compound and controls, test system, study design or study performance

### SciRAP Methodological Quality Criteria per Evaluation Domain

#### Test compound and controls

1. The chemical name or other identification, such as CAS-number, of the test compound was given
2. The purity of the test compound was stated or is traceable according to information given regarding manufacturer and lot/batch number. In case of mixtures, the composition of different constituents was stated
3. An appropriate solvent (vehicle) was used that is not expected to interfere with the results of the study at the concentration used
4. A solvent (vehicle) control was included
5. An appropriate positive control was included, and the expected result was observed from this treatment

#### Test system

6. A reliable and sensitive test system (e.g., cell line/cells/tissue/organ/embryo/sub-cellular fractions) with metabolic competence, if relevant, was used for investigating the test compound and endpoints
7. Conditions for cultivation and/or maintenance of the cell line/cells/tissue/organ/embryo/sub-cellular fractions (incubation temperature, humidity, CO2 concentration, media used, number of cell passages, control of contamination) were appropriate

#### Administration of the test compound

8. The duration of exposure was suitable for the test system and investigated endpoints
9. The concentrations used were suitable for the test system and investigated endpoints
10. The test conditions during and after exposure to the test compound were suitable (media and serum used, cell density, incubation temperature, humidity, CO2 concentration)

#### Data collection and analysis

11. Reliable and sensitive tests and/or analytical methods were used for investigating the endpoints
12. Sufficient numbers of replicates or repetitions of the experiment were used to generate reliable and valid results
13. Measurements were collected at suitable time points in order to generate sensitive, valid and reliable data
14. Cytotoxicity was measured and the test compound did not cause cytotoxicity that significantly affected the results
15. The statistical methods were clearly described and do not seem inappropriate, unusual or unfamiliar

#### Other

16. Are there any other aspects of study design, performance or reporting that influence reliability?

### SciRAP Relevance Item per Evaluation Domain

#### Test compound

1. The identity of the tested substance

#### Test system

2. The test system used

#### Endpoint

3. The endpoint studied

#### Concentration

4. The concentrations used

### Numerical Score for Reporting Quality and Methodological Quality

The SciRAP tool calculates a numerical score for reporting quality and methodological quality based on the formula below.

$$\mathrm{SciRAP}\;\mathrm{score}=\frac{F+(PF^{*}0.5)}{T}*100$$

F is the number of “fulfilled” criteria, PF is the number of “partially fulfilled” criteria, and T is the total number of criteria, excluding criteria that have been removed. The calculation of the score takes into account the weight attributed to individual criteria, i.e., each criterion is multiplied by its weight.
